# Lower back pain amongst medical trainees in clinical rotations: implications for choosing future career regarding medical practice

**DOI:** 10.3389/fpubh.2024.1412010

**Published:** 2024-11-05

**Authors:** Samuel Hakiranuye, Frank Kiwanuka, Daniel Asiimwe, Jussi P. Posti, Herman Lule

**Affiliations:** ^1^Department of Surgery, Kampala International University, Kampala, Uganda; ^2^Department of Nursing Sciences, University of Eastern Finland, Kuopio, Finland; ^3^Department of Surgery, Kabale University, Kabale, Uganda; ^4^Department of Neurosurgery and Turku Brain Injury Center, Neurocentre, Turku University Hospital and University of Turku, Turku, Finland; ^5^Injury Epidemiology and Prevention (IEP) Research Group, Turku Brain Injury Centre, Turku University Hospital and University of Turku, Turku, Finland

**Keywords:** occupational safety, backache, pain, medical career, job exit, occupational health, Africa

## Abstract

**Background:**

Low back pain (LBP) is an increasing concern amongst medical students. There is a dearth of publications regarding how the occurrence of LBP impact medical trainees’ career decisions.

**Objective:**

To determine: (i) the point and annual prevalence of LBP amongst Ugandan medical students, (ii) its associated factors, and (iii) whether the experience of LBP during clinical rotations influence medical students’ career choices regarding medical practice.

**Methods:**

A multi-center cross-sectional study of 387 randomly selected clinical-phase students was conducted in three Ugandan medical schools, during 17th January to 10th March 2023. Proportions of participants with current and 12-months history of LBP were computed as well as odds for career prospects. We performed binary logistic regression models to determine factors associated with LBP at 95% confidence interval regarding *p* < 0.05 as statistically significant.

**Results:**

The response rate was 100%. Participants’ mean age was 24.7 ± 3.2 years of which 66.2% (256/387) were males. The point and annual prevalence of LBP was 52.5% (203/387) and 66.1% (256/387) respectively. Age [OR 1.23, 95% CI (1.03–1.47), *p* = 0.02], time spent sitting per day [OR 1.08, 95% CI (1.06–1.3), *p* < 0.01], perceived influence of LBP on future medical career [OR 4.75, 95% CI (1.87–12.06), *p* < 0.01] were the significant predictors of LBP. LBP interrupted the students’ learning for at least 6.8 ± 12.8 h in 42.4% of participants. Nearly half of participants affirmed that their LBP experience would influence their career prospects. Based on their LBP experiences, trainees ruled out surgery 51.5% (172/334), obstetrics/gynecology 29.6% (99/334), paediatrics 18.3% (61/334), and internal medicine 17.7% (59/334) as their future career specialties. The proportion of trainees that would not consider surgical as opposed to medical disciplines were 81.1% vs. 36.0%, respectively, (*p* < 0.001).

**Conclusion:**

The high prevalence of low back pain among medical students impacts their choices of future medical career with an aversion towards specialization in surgical disciplines. This has far-reaching implications on the disparities in specialist physician health workforce in Low-middle-income countries.

## Introduction

Low back pain (LBP) is the single most contributor to musculoskeletal disability, reduced productive working hours and work absence (1). Ultimately, LBP results in 8.1% of all-cause years lived with disability (2). For instance, LBP affected 619 million (one in every 13) people in 2020 and is projected to affect 843 million people globally by 2050 (2). The global point and annual prevalence of LBP ranges from 12–33% to 22–65%, respectively, (3) affecting both low-middle-income (LMICs) and high income countries (HICs) (4). However, the projected increase in LBP burden is expected to be highest in LMICs as a result of evolving population aging and use of inefficacious costly treatments (1). In HICs, including those in Americas, Europe and Western Pacific; a recent systematic review estimated the pooled annual rate of hospitalization due to LBP at 0.6–5.7%; which imply a pooled annual direct and indirect total cost of US$10144 per patient (4).

On the other hand, in LMICs, including those in Asia, South America and Africa; the annual pooled rate of hospitalization due to LBP ranged between 13.4–18.7% resulting in an annual total cost of US$1226 per patient (3). In a recent prevalence-based cost of analysis study in South Africa, the total annual average direct cost due to acute and chronic LBP were US$99 and 1516, respectively, (5). It has been difficult for researchers to compare the burden of LBP based on pooled global prevalence of hospitalizations due to heterogeneity of studies (6) but the limited human resources for health, constrained health care budgets and inadequate prioritization of LBP research aggravates the morbidity of LBP in LMICs compared to HICs (7). According to the critical evaluation of 22-years trend in LPB-related publications, there was compelling evidence to suggest disparities, where HICs preceded LMICs yet the latter is home to 85% of the world’s population (8).

LMICs such those in Africa have the least developed ergonomic technologies to prevent work-related LBP thus should be at the forefront of high quality population-level research aimed at risk detection, early diagnosis, and treatment of LBP but there is a lack funding (7). According to a systematic review and meta-analysis by Moris et al. (9), the pooled point and annual prevalence of LBP in Africa were 39 and 57%, which are higher than the global point (18.3%) and annual (38.5%) prevalence of LBP, respectively, (10). Synthesized evidence suggest that 80–90% of the African population’s work is physically demanding; entailing heavy lifting; which together with socioeconomic constraints, coexisting malnutrition and tuberculosis of the spine underpin the high burden of LBP in Africa (11). Moreover there is a growing burden of LBP in the young African population with a point prevalence of up to 58% amongst African adolescents (9), which counteracts the United Nations’ mission of healthy aging (2).

Recent studies have demonstrated that other than pathological causes, non-specific LBP ascribed to lifestyle factors and work environments contribute to 90% of cases (12). Systematic reviews demonstrate that working in healthcare settings is considered one of the top 10 risky occupations for developing LBP (13). In Africa, the burden of LBP amongst health workers is aggravated by the lack of assistive devices to move patients with disabilities; largely attributable to: limited production, low quality, and prohibitive costs of such devices (14). As such, standard ergonomic work practices are embryonic which contributes to higher burden of LBP amongst health practitioners in Africa (12, 15). For instance, in a recent systematic review by Kasa et al. (15), the pooled point prevalence of LBP amongst African nurses was 64.1% (95% CI 58.7–69.5). Prolonged standing, lifting and transfer of patients, repeated bending or twisting and working in awkward postures are the main risks for LBP amongst African health workers (16). These factors have been previously identified as proxy for occupational ergonomic exposure to LBP (2). Indeed a previous cross-sectional study in Nigeria showed that nurses who self-reported the above factors at their work environment also had intentions to change their workplaces or quit the nursing profession (16).

Uganda is one of the African countries in the low-income category where researchers have documented LBP as an occupational hazard amongst its qualified health workers, with a point prevalence of 39.6% (12). For qualified Ugandan health care providers, working conditions and occupational hazards are closely monitored by the Ministry of Health as mandated by the Labour laws. However, medical students largely rely on their training institutions to address issues related to work organization, management, and working conditions despite their limited knowledge of occupational hazards. While manual patient handling, repetitive bending or twisting, and extended working hours are known contributors to LBP among licensed Ugandan health workers (12), the extent of ergonomic risks related to these activities has not been thoroughly examined among Ugandan medical trainees who form 75% of Uganda’s health workforce (17), despite emerging global evidence which demonstrate considerably high prevalence of LBP amongst students (18–20). In a systematic review of 16 studies which evaluated 7072 students, the annual prevalence of LBP amongst nursing and medical students was 44% (95% CI 27–61) and 53% (95% CI 44–62) respectively, whereas the incidence rate of LBP amongst nursing students ranged from 29 to 67% but studies never reported the incidence rate amongst medical students (21). Being in a final study year (psychosocial stress and anxiety), female sex (21), long study hours and sedentary lifestyle are some of the documented factors contributing to LBP amongst medical trainees (19).

Evidence show that LBP has influence on the employees’ decision to exit paid employment especially amongst lower grade employees (22). Globally, medical trainees and interns rank the lowest in seniority withing their work environment, thus early detection of those potentially at risk of developing LBP and their intent to exit or change their career paths are critical to prevention policy aimed at retaining the employee pool in the labor market. The aim of this study was to determine: (i) the point and annual of LBP among Ugandan medical students, (ii) the factors associated with it, and (iii) whether the experience of LBP during clinical rotations influenced medical students’ career choices regarding medical practice.

## Materials and methods

### Study design

A multi-institutional cross-sectional online self-administered survey questionnaire was sent to 387 randomly selected clinical year medical students who endorsed an electronic consent form to participate in the research during 17th January to 10th March 2023. The cross-sectional design was suitable due to the lack of robust electronic medical records in Uganda which prohibited the use of retrospective cohorts. We defined LBP as pain in the posterior aspect of the body that lasted at least a day or more in the area between the lower margin of 12th rib and the lower gluteal folds with or without limb involvement in accordance with previous studies (2). We used the current and 12-months recall periods to minimize recall bias in accordance with previous studies (12).

### Study population and settings

The survey was conducted in three medical schools and their respective teaching hospitals in Uganda (two public and one private), including: Mbarara University of Science and Technology located in the city of Mbarara; Kabale University located in Kabale city, and Kampala International University with campuses located in Kampala capital city and Ishaka. In total, these medical schools have 10 affiliated teaching hospitals where students undertake clinical rotations. In the academic year that preceded the survey, these medical schools in total boosted 4,000 medical students both from various parts of the country and from abroad. The participants were undergraduate students enrolled in Bachelor of Medicine and Bachelor of Surgery Degree (MBChB) hereafter being referred to as medical students. To obtain the MBChB degree in Uganda, candidates who have completed two preparatory college years, and have succeeded in biological sciences are enrolled to study for at least 5 years plus one additional year of pre-licensure supervised internship. The first 2 years of medical training are preclinical whereas years three, four, and five are clinical. The clinical year students attend lectures, seminars, bed-side teaching ward rounds, and assist in surgical operations and dispensing medication under supervision. Further, the students perform clinical clerkships, case writeups, and are paired with intern doctors to attend medical and surgical emergencies which contributes to their logbooks.

### Eligibility criteria

The university medical schools were purposively selected to represent a mixture of public and private institutions. All medical students in their third, fourth and fifth (final) year of study who were undertaking their clinical rotations at the respective medical schools and affiliated teaching hospitals had a chance to participate in the study. These clinical-phase students were selected on the basis that previous studies identified patient manual handling amongst healthcare providers as a risk for LBP (12) of which trainees in clinical rotations perform such tasks. We excluded students who were designated as non-attending for the academic semester during which the study was conducted; as well as those with documented history of physical injuries resulting from falls, traffic crash and congenital spine deformities that could lead to LBP (23).

### Sample size determination

At the time of the study, the total population of medical students in the three medical schools was 4,000, considering all academic years. The sample size was determined using a hypergeometric formula for known small populations,


$$\left(n\right)=\frac{NZ^{2}PQ}{\left\{E^{2}\left(N-1\right)+Z^{2}PQ\right\}}$$

Where (*n*) = required sample size; N = population size (4,000); E = value setting accuracy of sample proportions (0.05); *Z* = value for the level of confidence (1.96) at 95% confidence interval; P = proportion of medical students that suffered LBP, *Q* = (1-P). Since these proportions were unknown, P and Q were assumed to be 50%, which by substitution, yielded 351 participants. We added 10% to cater for non-response and obtained a total sample size of 387 students. In this exploratory study that did not aim at establishing causal inference, it was deemed unnecessary to compute a sample size as way of demonstrating a valid association between each covariate with LBP.

### Sampling procedure

Following administrative and ethical clearance, a random sample of 387 participants was drawn without replacement from a pool of 1100 medical trainees who were registered as attending in the clinical disciplines (third, fourth, and fifth year students), using XLSTAT software for windows (XLSTAT add-on statistical software, 2023. Lumivero, Denver, USA). For equal representation, the number of sampled participants were proportional to the total number of medical students in clinical years at each university. The proportion (P) depending on the population of students in clinical years (Nc), was calculates as:


$$P=\mathrm{Sample}\;\mathrm{size}\;\left(n\right)\times \frac{100}{N_{c}}=387\times \frac{100}{1100}=35.2\%$$

Thus 35.2% of each medical school’s clinical-year students were studied, which meant 209 participants from Kampala International University, 89 from Kabale University and 88 from Mbarara University of Science and Technology.

### Study procedure

Participants were sent weekly email reminders to complete the online google form survey though their class representatives and university secretaries. Each participant completed an electronic consent form followed by a pre-tested authors’ questionnaire modified from Aleku et al. (12), which demonstrated a tests-re-test reliability coefficient of 0.9. The questionnaire (Supplementary material S1) captured demographic variables (age, sex, year of study); personal (alcohol consumption and cigarette smoking); and work-related factors (current and most recent clinical disciplines, ergonomics of work environment such as time spent standing, sitting, lifting, bending, or transferring patients as well as career pursuits). These variables had been documented to influence LBP amongst medical students in previous studies (18–21).

### Ethical considerations

This study followed the Uganda National Council for Science and Technology (2014) guidelines on research involving humans as research subjects. Ethical clearance was obtained from the Research and Ethics Committee of Kampala International University, Faculty of Clinical Medicine, and Dentistry (Ref: BMS/11775/173/DU). All participants signed a predesignated electronic informed consent form prior to participation.

### Data analysis

Descriptive analysis of the sample characteristics were presented as means, standard deviation, percentages and frequencies. Other descriptive statistics were performed according to each objective.

A predefined assessment of LBP was included in the instrument used for data collection. We computed the percentage of participants with LBP by dividing the number of people with LBP by the total number of participants and multiplying by one hundred.

#### Factors contributing to LBP

Participants rated a range of factors affecting LBP in their work environment on a scale from 0 to 5. These factors included work schedule, posture, and fatigue, among others as identified in previous studies (12). In addition, there were open ended options to detail other factors which participants regarded as important. We ranked the overall impact of these factors based on their average scores. Factors that were signficant at the bivariate level of analysis were further examined in a binary logistics regression.

#### Impact on career choices

The study assessed whether LBP affected trainees’ future career decisions. It looked at how many trainees said their LBP experience influenced their specialty choices and compared the proportions of those considering surgical versus medical fields using Chi-square test of independence. All analyses were performed in R statistical environment (V4.3.1 R Core Team 2023). All tests were two tailed and considered significant at 95% confidence interval, when *p* < 0.05.

## Results

All the 387 returned the questionnaire (response rate 100%). The mean age of participants was 24.7 years (SD = 3.2). Majority were males 66.2% (*n* = 257) and had completed their fourth year of medical training (38.5%, *n* = 149). Only 1.6% (*n* = 6) of participants reported that they were currently smoking cigarettes. Almost a third of participants indicated that they were currently consuming alcohol (28.4%, *n* = 110). More than half of the participants were in a private medical training institution (Kampala International University). At the time of the survey, most participants were undertaking clinical rotations in surgery 25.1% (*n* = 97), and internal medicine 22.7% (*n* = 88) (Table 1).

**Table 1:** Sociodemographic and behavioral characteristics of study participants.

Variable	Category	Frequency (n)	Percentage (%)
Sex assigned at birth	Male	257	66.2
	Female	130	35.5
Age	Median (SD)	24.7 (3.2)	
Institution	KIU	209	54.1
	MUST	88	22.8
	Kabale University	89	23.1
History of ever smoking cigarette	No	361	93.0
	Yes	27	7.0
History of current cigarette smoking	No	382	98.5
	Yes	6	1.5
History of alcohol consumption	No	71.6	28.4
	Yes	278	71.6
Year of study	Year three	106	27.3
	Year four	150	38.7
	Year five	132	34.0
Current clinical rotation	Surgery	97	25.1
	Internal medicine	88	22.7
	Pediatrics	69	17.8
	Obstetrics and gynecology	60	15.5
	Dermatology	36	9.3
	Psychiatry	26	6.7
	Anesthesia	13	3.4
	Ear Nose Throat	7	3.4
	Ophthalmology	7	3.4
Are you currently experiencing low back pain lasting at least one day?	No	184	47.5
	Yes	203	52.5
Have you ever experienced low back pain as a medical student in the past 12 months?	No	131	33.9
	Yes	256	66.1
Which clinical rotation were you at the first onset of low back pain? (N = 296)	Surgery	106	35.8
	Obstetrics and gynecology	103	34.8
	Internal medicine	89	30.1
	Pediatrics	56	18.9
	Anesthesia	6	2.0
	Psychiatry	4	1.4
	Ophthalmology	3	1.0
	Dermatology	3	1.0
Years of clinical exposure	Mean (SD)	2.2 (2.0)	
Duration spent in current clinical rotation in months	Mean (SD)	1.9 (3.8)	
Duration spent other clinical rotations in months	Mean (SD)	2.0 (1.3)	
Time spent lifting per day in current clinical rotation (Hrs.)	Mean (SD)	1.4 (5.3)	
Time spend bending per day in current clinical rotation (Hrs.)	Mean (SD)	1.6 (1.7)	
Time spent transferring dependent patients per day in current clinical rotation (Hrs.)	Mean (SD)	1.1 (1.4)	
Time spent sitting per day in current clinical rotation	Mean (SD)	4.8 (6.0)	
Time spent standing per day in current clinical rotation	Mean (SD)	4.8 (2.5)	

KIU, Kampala International University, MUST, Mbara University of Science and Technology.

### Prevalence of low back pain

**Figure 1:**
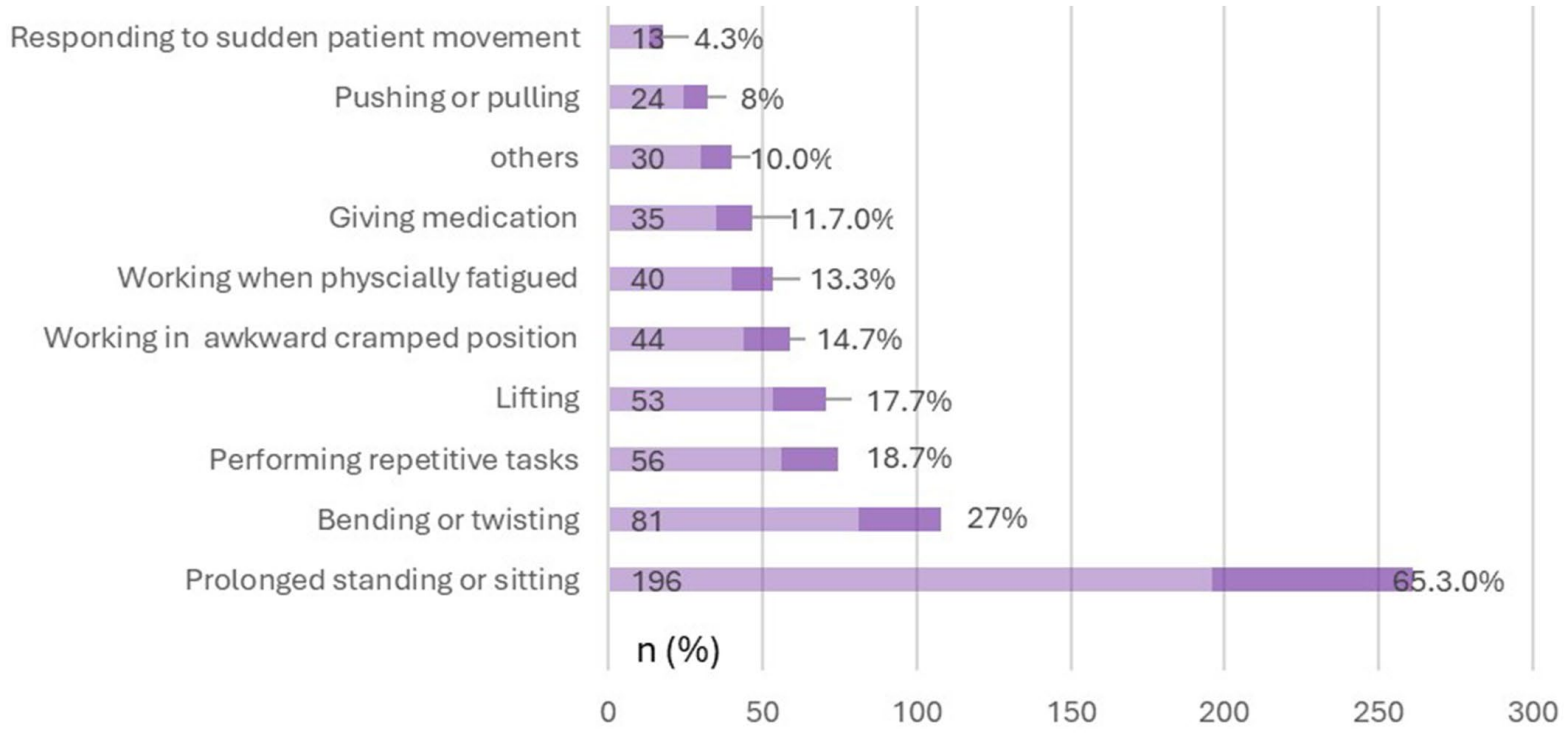
Detailing participants’ activity at the onset of their low back pain (*N* = 300).

**Figure 2:**
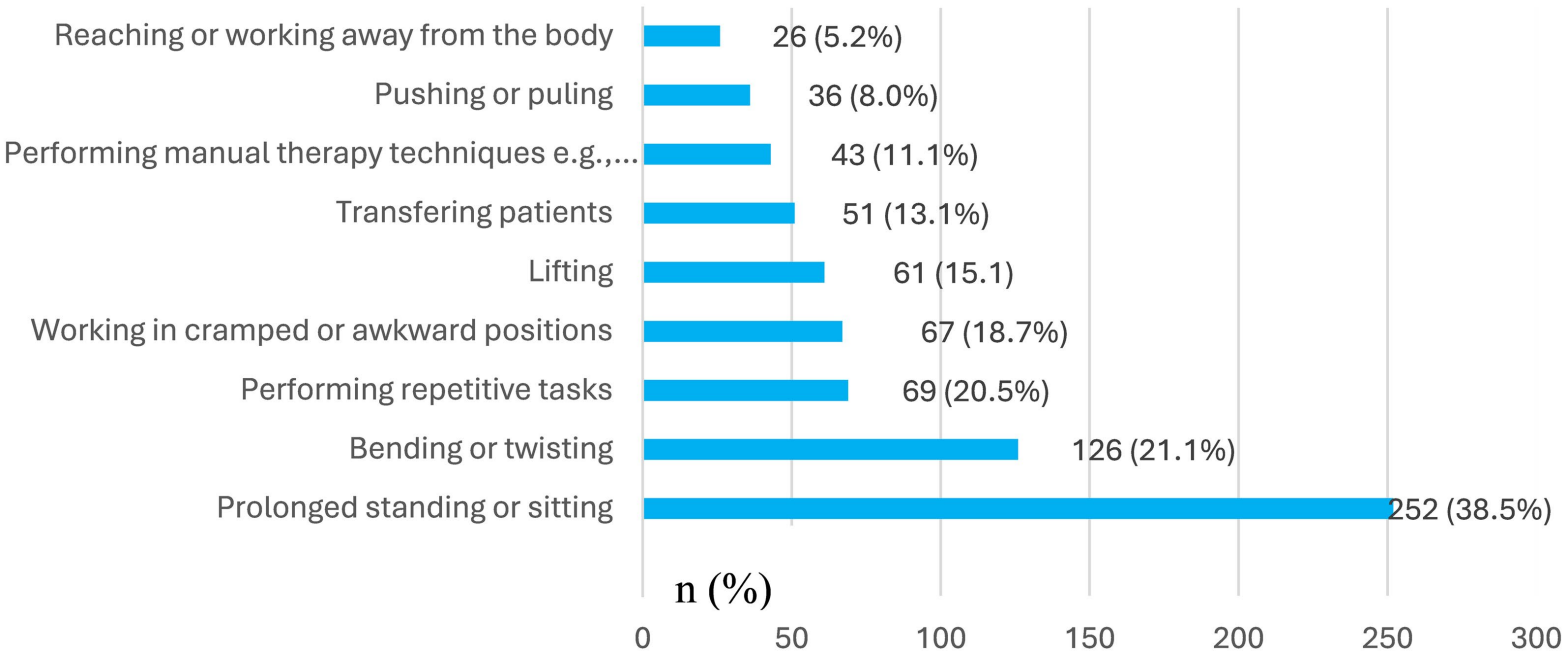
Participants’ view of activities that triggered recurrence of their low back pain (*N* = 327).

**Figure 3:**
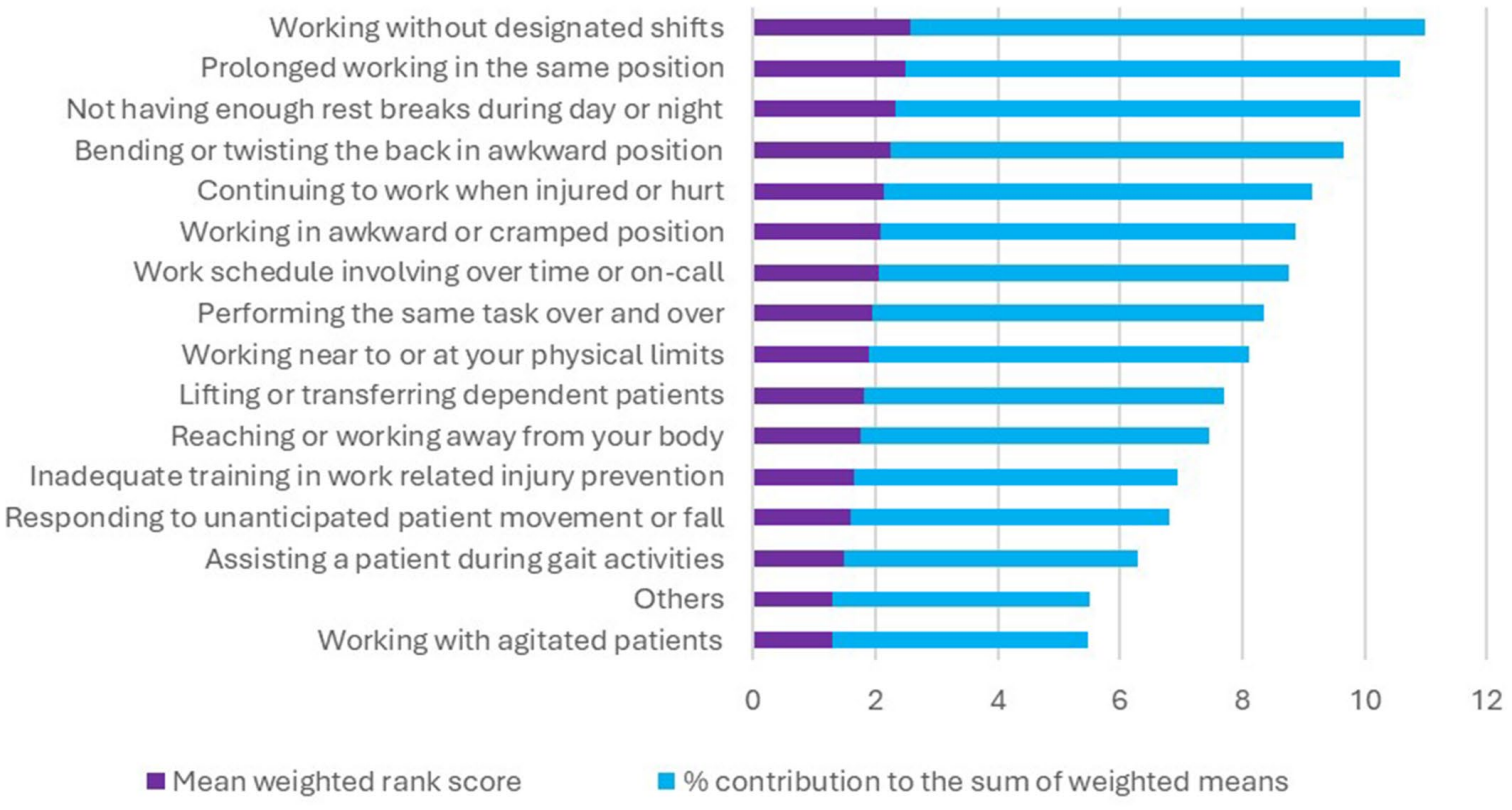
Weighted mean rank scores for exposure to low back pain during clinical clerkship.

Of the 387 participants, 52.5% (*n* = 203) suffered LBP at the time of the survey whereas 66.1% (*n* = 256) had suffered LBP within the past 12-months. Moreover 42.4% (164/387) reported that LBP ever interfered with their class work or ward session for an average duration of 6.8 h (12.8 SD). Most participants were rotating in surgery 35.5% (106/296), obstetrics and gynecology 34.8% (103/296) at the onset of their LBP. The majority 65.3% (196/300) of respondents attributed prolonged standing or sitting as the main activity when they noticed their first episode of LBP (Figure 1) whereas 77.1% (252/327) identified prolonged standing or sitting as the trigger for recurrence of their LBP (Figure 2). The highest ranked perceived exposure to LBP at the participants’ work environment was working without designated shifts (Figure 3).

### Factors associated with low back pain

The one sample students t-test revealed that the mean difference of those with and without current LBP significantly differed across participants’ age *t*(387) = 153, *p* < 0.001, duration spent in clinical rotation *t*(387) = 20.6, *p* < 0.001, time spent while: standing *t*(387) = 20.6, *p* < 0.001, sitting *t*(387) = 20.6, p < 0.001, bending *t*(387) = 20.6, *p* < 0.001, lifting *t*(387) = 20.6, *p* < 0.001 and transferring patients *t*(387) = 20.6, *p* < 0.001 per day during the clinical rotation. There were no statistically significant differences with respect to current experience of LBP across sex [*X*^2^ (1, *N* = 387) = 0.67, *p* = 0.41], and participants’ current clinical rotation LBP [*X*^2^ (7, *N* = 387) = 10.2, *p* = 0.178]. Current LBP was associated with history of “ever” smoking of cigarette [*X*^2^ (1, *N* = 387) = 4.357, *p* = 0.037] but not with history of “current” cigarette smoking [*X*^2^ (1, *N* = 387) = 1.029, *p* = 0.310] or history of alcohol consumption [*X*^2^ (1, *N* = 387) = 0.005, *p* = 0.946].

Binary logistic regression analysis of factors associated with LBP showed that age [OR 1.23, 95% CI (1.03–1.47), *p* = 0.02], time spent sitting per day during the current clinical rotation [OR 1.08, 95% CI (1.06–1.3), *p* < 0.01], and perceived influence of LBP on future medical career [OR 4.75, 95% CI (1.87–12.06), *p* < 0.01] significantly predicted LBP. The best fit model demonstrated that predictors were associated with 49.7% of occurrence of LBP (adjusted *R*^2^ = 49.7) as shown in (Table 2).

**Table 2:** Binary logistic regression analysis for predictors of LBP.

Variables	Estimate	Standard error	*p*-value	OR	95% CI	
Age	0.43	5.51	0.02*	1.23	1.03	1.47
Time spent in current clinical rotation (months)	−0.36	0.18	0.3	0.95	0.85	1.05
Time spent lifting per day in current clinical rotation (hours)	−0.41	0.42	0.32	0.71	0.43	1.16
Time spent bending per day in current clinical rotation (hours)	0.24	0.24	0.30	1.07	0.80	1.44
Time spent transferring patients per day in current clinical rotation (hours)	0.21	0.39	0.58	0.95	0.62	1.45
Time spent standing per day in current clinical rotation(hours)	0.21	0.15	0.15	0.82	0.72	0.92
Time spent sitting per day in current clinical rotation (hours)	−0.22	0.10	<0.01*	1.08	1.06	1.3
Perceived influence of LBP on future medical career	0.10	0.50	<0.01*	4.75	1.87	12.06

OR, Odds Ratio; CI, Confidence Interval; *Statistically significant at *P* < 0.05.

When asked whether experiencing LBP during clinical rotations would influence their intent to specialize in a particular discipline for a medical career, 49.6% (192/387) of participants indicated “yes.” The odds of disagreeing were lower amongst those who were currently suffering from LBP compared to those were not, i.e., [OR 0.474, 95% CI (0.382–0.589)] vs. [OR 2.309, 95% CI (1.808–2.950)], *p* < 0.001. The odds of disagreeing were also lower amongst those who had experienced LBP in the past 12 months compared to those who had not, i.e., [OR 2.598, 95% CI (1.872–3.606)] vs. [OR 0.631, 95% CI (0.542–0.735)], *p* < 0.001.

Based on their perceptions and LBP experiences, medical students indicated they had no intent to pursue surgery (including orthopedics, physical therapy, and trauma rehabilitation) 51.5% (172/334), obstetrics and gynecology 29.6% (99/334), paediatrics 18.3% (61/334), internal medicine 17.7% (59/334). The proportion of trainees that would not consider surgical as opposed to medical discipline were 81.1% vs. 36.0%, respectively, and the difference in proportions was statistically significant [*X*^2^ (1, *N* = 334) = 140.6, *p* < 0.001].

## Discussion

This study aimed at determining the point and annual prevalence of LBP amongst Ugandan clinical-year medical trainees, its associated factors, and whether the experience of LBP during clinical rotations influenced the learners’ career choices regarding medical practice. The point and annual prevalence of LBP was found to be 52.5 and 66.1%, respectively. Previous studies have reported the point and annual prevalence of LBP amongst medical students as: 37.8 and 80.4% in Tunisia (19); 25.6 and 63.3% in Bangladesh (18); 14.4 and 66.8 in Brazil (24); 17.2 and 59.5% in Serbia (25); 10.1 and 44.9% in Saudi Arabia (26); 42.1 and 72.1% in France (20) respectively. Thus, although the 12-month prevalence was comparable, the point prevalence in the present study was slightly higher than reported in previous studies. The difference could result from variation in inclusion criteria, having limited the present study to trainees in clinical years.

Regarding the predictors of LBP, we found that age (*p* = 0.02) played a role in contrast to previous studies (26). Researchers in Tunisia (19) and France (20) have argued that age itself might not be a predictor of LBP amongst medical students but rather the year of study, which changes with increasing psychosocial stress as one approaches the terminal clinical years (21, 27). However, our study did not support this notion as neither the year of study (*p* = 0.09) nor the time spent in the clinical rotation (*p* = 0.3) as a biomarker for duration of exposure were associated with LBP. Although some scholars documented 1.8 times frequence of LBP amongst female medical students (21), we did not observe this relationship in congruity with other studies (26).

Further, we found that time spent sitting per day during clinical rotations was associated with LBP (*p* < 0.01). Moreover, most participants (77.1%) identified prolonged sitting during classes and prolonged standing during ward rounds as the key triggers for recurrence of their LBP, with average sitting and standing time of 4.8 ± 6.0 h and 4.8 ± 2.5 h, respectively. Other scholars have identified sedentary lifestyle as a contributing factor to LBP amongst medical trainees. In a cross-sectional study of 207 medical students in Bangladesh, it was established that sitting more than 6 h per day was a predictor of LBP (18). In another study of 300 medical trainees in Saudi Arabia (26), researchers found that those who did not do physical exercises were three times more likely to report LBP whereas those who spent more than 8 h sited had 5.6 times increased risk of LBP. According to a French study of 1243 medical students, it was found that trainees who walked at least 30 min per day and performed weekly vigorous exercise were less likely to report LBP (20). Moreover LBP had a negative impact on students’ day work performance, and on their quality of sleep which created a vicious cycle of LBP in their personal lives (20).

To minimize this undesirable effect of sedentary lifestyle during medical training, experts in USA advised on the inclusion of medical trainees as longitudinal exercise co-instructors for patients with or at risk of LBP, with room to incorporate the students’ suggestions for the physiotherapy and medical programme improvement (28). Moreover, an inclusion of moderate levels of physical activity within the trainees’ work environment had been suggested in a previous systematic review that evaluated the association between physical activity and LBP (29), in accordance with the World Health Organization’s holistic non-invasive approach to LBP which contextualizes individuals’ unique workloads (30).

However, the busy schedule of medical students often precludes them from exercise. In a cross-sectional survey of 377 Saud Arabian medical students, 45.4% did not engage in any physical activity other than walking due to time constraints (31), which imply the necessity for innovative physical activity plans that accommodate the unique busy demands of medical trainees. Moreso, research has shown that even physiotherapy students do not practice what they preach; often fail to fulfil their daily exercise demands and self-back care, leading to LBP. For instance, a Brazilian study found that physiotherapy students were 2.5 times more likely to report LBP compared to medical students (24), whereas in the present study, 13.1% of our participants attributed their LBP to performing manual techniques such as massage and assisting patients to ambulate during their assignment to the physiotherapy unit. To mitigate the possibility of future back health practitioners becoming patients themselves, Australia’s clinical care standards guidelines advocates for introduction quality indicators to enable care providers measure how well they implement prevention and early treatment strategies for both patients and health providers (32). This approach should be used for medical students who report their first episode of LBP since a mushrooming epidemic of poor care has been identified in the Lancet series as the most critical aggravating factor for LBP burden (2).

Finally, we found that perceived influence of LBP experience on future medical career choices (*p* < 0.01) was a predictor of LBP. Moreover, our findings showed that the experience of LBP during clinical rotations would influence the career choices of nearly 50% of medical trainees and consequently more than 50% ruled out surgical disciplines as potential choices to consider for specialization. This aversion is likely due to the prolonged standing associated with surgical disciplines as undergraduate clinical students scrub-in to observe and or assist during major theatre operations. Each of such operations in surgical disciplines such as general, orthopaedics and neurosurgery could last as long as 2 h or more, and while this might the case for medical trainees globally, the situation is worse in LMICs as from the authors’ experience; a typical theatre list could have up to four to six patients due to the overwhelming surgical patient backlogs in lieu of the low doctor-patient ratio in LMICs (33). Also, it is the case that for medical students who are not scrubbed-in to actively assist in surgical operations due to limited theatre space and infection control protocols would have to sit and watch the real-time broadcast or recorded videos for advanced operations which contributes to their sedentary screen time. Indeed, prolonged siting and standing were the most cited triggers of onset and recurrence of LBP amongst medical students in the present study.

Overall, the results of the present study have threefold policy implications. First of all, although LBP is principally a non-surgical disease (2), referral for interventional procedures and surgical approaches form an integral part of progressive LBP treatment that is nonresponsive to other therapy (32, 34). Moreover the authors’ experience in Africa and Europe is that patients with LBP would typically visit a general practitioner or occupational physical therapist followed by a neurosurgeon or orthopaedic spine surgeon in that order which corroborates with evidence from the USA that demonstrates LBP as the third leading cause of surgical specialty consultations (35). However, compelling evidence suggest that LMICs already have an unmet need for 143 million surgical procedures per year to adequately prevent disability including that due LBP as the current surgery case volume in these countries is far below 5000 procedures per 100,000 population per year recommended by the Lancet Commission on Global Surgery (33). Thus the intent not to consider surgical specialties for career choices amongst medical students is worrisome in Uganda which boosts of only one surgeon per 100,000 persons (17). Moreover, how this will affect future human resource capacity for LBP care within the context of specific causes such as due to neural compression and degenerative lumbar spine diseases should be an emerging area of research interest as the country already suffers reduced numbers of surgical professionals, low-operative volumes and poor access to surgical, obstetrics, and anesthesia care (36).

More specifically to put our results into local context, an earlier study of 418 medical students in seven Ugandan medical schools established that the majority (52.6%) would consider a career in internal medicine due to the donor funding bias towards infectious diseases and an opportunity to get “time-off clinical work” for research (37). Moreover in another survey of 251 final year Ugandan medical students which assessed their career intentions after graduation, it was found that due to their presumed safer ergonomic working conditions abroad, 44.6% planned to emigrate from Uganda to USA, UK or South Africa whereas 11.2% intended to abandon the health sector to join business, agriculture or politics due to the overwhelming workload and risky working environment amidst low wages (37). Thus, our findings have policy implications for sustainability in production and maintaining a pool of future LPB care givers through medical training locally and globally. Accordingly, software developers, occupational health engineers, and health educators should devise low-cost simulations as adjunct to traditional clinical rotations in LMICs to minimize the situations that predispose medical trainees to LBP in their physical work environments. Evidence show that 11–30% of clinical training time could be replaced with simulated placements without endangering patients and learners (38, 39).

Secondly, our study revealed that LBP had interrupted the learning process during class work or ward sessions for at least 6.8 h (12.8 SD) in 42.4% of participants. LBP is a known cause of sickness absence from work, with an estimated prevalence of annual absence from work ranging from 12.5% in the UK, 9% in New Zealand to 32% in Ireland (40). In a recent global burden of disease study, it was established that LBP had led to an average absence from work of 100 days in Brazil and 10 days in the USA per person per year (2). Moreover, according to a systematic review by Wynne-Jones et al. (40), up to 32% of individuals who suffer an episode of LBP are not able to return to work within a period of 1 month and 6.7% may not return within 6 months from the time of onset. For the case of LMICs, absence from work implies a double loss as learners miss from their studies while at the same time, this strains the skeleton health workforce as medical trainees form an integral part of human resource for health in rural African settings (17). For instance, as demonstrated in this Ugandan study, a portion of medical students are routinely assigned to the physiotherapy unit as part of their surgery rotation to assist with physical therapy techniques not only to beef up the skeleton staffing but also to address the inequalities in access to back health practitioners.

Lastly, the fact that most of our participants cited prolonged sitting, standing, and working without designated breaks during shifts as triggers for their LBP recurrence during clinical rotations deserves attention. In a systematic review by Wong et al. (21), it was found that students who had prior history of LBP were 3.5 times more likely to develop recurrence within 1 year compared to those with no prior history, emphasizing the need to holistically address potential barriers to full recovery. Medical trainees are the future generation frontline health workers for LBP care. The current ergonomic clinical working conditions of medical trainees in LMICs such as lifting and transferring patients manually have potential to predispose them to LBP, with profound consequences on their retention in clinical practice after graduation. According to a 28-year follow-up of 8665 British Whitehall II cohort study (22), it was found that employees who experienced recurrence of LBP were 1.5 times more likely to exit from their paid employment due to health related conditions compared to those who did not report LBP, having controlled for other socioeconomic modifiers. Moreover it’s the lower grade and middle grade workers who were more likely to exit the workforce (22). In medical profession, often medical students and interns have the lowest rank amongst the LBP care frontline workers. At the time of the study, there were only 500 physiotherapists serving Uganda’s 50 million population, as medical trainees including interns and residents contribute to 75% of the country’s health workforce (17). Thus, it is imperative to address the events that trigger LBP to reoccur to prevent early retirement or change of career amongst medical trainees. A mixed methods study of 57 medical trainees in England found that LBP emerged from all work cycles of “the students’ life” such as lectures, seminars and clinical ward rounds which demanded various body postures including sitting, standing, bending (41) but inadequate breaks between tasks has been singled out as an important contributor to LBP in the students’ population (18).

### Study strengths and limitations

In terms of strengths, this one of the few multi-institutional studies in Africa which have probed trainee medical professionals’ career intentions based on their LBP experience during clinical rotations and have explored their own voices regarding the perceived exposure to LBP in clinical settings. In addition, the high response rate and random sampling improved reliability and generalizability of our findings to train health professionals. On the contrary, there were limitations that are worth acknowledgement. First, we collected the data from self-reports both regarding the occurrence of LBP, its perceived triggers and effects on intended career choices which could raise concerns about recall and social desirability bias. However, self-reports, point prevalence and 12-months recall periods have been used in previous studies (12), and there no particular reasons to think that students would not correctly report whether they intend not to specialize in particular disciplines; besides, why would the career choices differ by LBP experiences during varying clinical rotations. After all, the experience of LBP is subjective as the absence or presence of pain is better expressed by the individual experiencing it, although the threshold could differ between individuals (29). Furthermore, this study focused mostly on the physical working conditions but to a lesser extent, psychosocial working conditions also play a role in LBP as evidenced from pooled analysis of studies in US, Japan and Norway (42). Lastly, uncertainly remains when using LPB experience to predict career choices in a cross-sectional study design as the trainees’ experiences could change over time, although based on a Danish study (43), and on a systematic review of LBP amongst medical students (21), its known that occurrence of LBP early in life due to strenuous work predicts its continuity or recurrence later in life.

## Conclusion

The point prevalence of LBP was 52.5% and was higher than reported in existing literature although the annual prevalence was comparable at 66.1%. The time spent sitting was the key modifiable factor associated with LBP. LBP interrupted the students’ learning for at least 6.8h in nearly half of the participants. Previous studies have primarily focused on determining the prevalence of LBP and its associated factors among medical students. In contrast, the present study further elucidates that experiencing LBP during clinical rotations significantly influences medical trainees’ career choices regarding medical practice. Notably, more than half of the trainees expressed no intention to specialize in surgical disciplines. Our findings have implications for future specialized human resource pool derived from medical trainees regarding LBP care and could worsen the existing specialty health care disparities in LMICs. Ergonomic restructuring, structured exercise programs blended with clinical simulations could minimize the situations that predispose medical trainees to LBP. Future studies should be long-term prospective cohorts that evaluate LBP at multiple time points to ascertain how this impacts the medical trainees’ career choices after graduation.

## Data Availability

The original contributions presented in the study are included in the article/Supplementary material, further inquiries can be directed to the corresponding author.
