# Supplementary material for: Lower back pain amongst medical trainees in clinical rotations: implications for choosing future career regarding medical practice
Source: Front Public Health. 2024 Nov 5;12:1412010. doi: 10.3389/fpubh.2024.1412010 (PMC11573755; doi:10.3389/fpubh.2024.1412010)
Supplement: Supplementary file 2 [file Data_Sheet_2.DOCX]

**Supplementary materia1: (questionnaire)**

This research will assess the prevalence and associated factors of low back pain among *medical students* and will establish how this might influence future career choices. Please answer the following questions. Where there are multiple choices, please circle/tick the correct response. Navigate from left to right before the next page.

**Section 1: Individual Factors**

1. Year of study: ………………….………………
2. Name of medical school ……………………………
3. What is your current age in years?.......................
4. Tick the sex assigned to you at birth.

MALE FEMALE OTHER

1. **History of Smoking and Alcohol Consumption**

| 1. Do you currently smoke cigarettes? | YES | NO |
| --- | --- | --- |
| 2. Have you ever smoked cigarettes for some time before? | YES | NO |
| 3. Do you often drink alcohol? | YES | NO |

1. **Shift Rotation Details.**

| 1. How many years have you been practicing in the hospital? |  |
| --- | --- |
| 2. Which discipline is your current clinical rotation? |  |
| 3. How long have you rotated in the current discipline? |  |
| 4. What other departments have you rotated in the last semesters? |  |
| 5. If yes, for how long have you spent in other clinical rotations altogether? |  |

**Section 2: Low back pain (LBP) History:**

**Definition: Knowing that low back pain (LBP)** is *pain lasting at least 1 day in an area between the 12^th^ribs and the gluteal folds:*

**7)**

| 1. Have you suffered from LBP currently within the past 3 months? | YES | NO |
| --- | --- | --- |
| 2. Have you ever suffered from LBP as a medical student in the past 12 months? | YES | NO |
| 3. Has low back pain ever interfered with your classwork / ward sessions?  If Yes, how long (in hours) …………. |  |  |
| 4. Which rotation were you doing when the lower back pain started? (answer only if you have ever suffered) |  | |

1. **Which activities cause your Low back symptoms to recur?** Please Tick all that apply (if any).

| 1. Bending or Twisting |  |
| --- | --- |
| 2. Lifting |  |
| 3. Maintaining a position for long periods of time e.g. standing, sitting |  |
| 4. Performing manual therapy techniques e.g. massage, mobilization |  |
| 5. Performing repetitive tasks |  |
| 6. Reaching or working away from the body |  |
| 7. Transferring patients |  |
| 8. Working in cramped/awkward positions |  |
| 9. Pushing or pulling |  |
| 10. Others. Please specify: |  |

1. **If you have ever visited a doctor and know your diagnosis (cause) of LBP, please tick/specify.**

| 1. N/A (No diagnosis) |  |
| --- | --- |
| 2. Degeneration |  |
| 3. Ligament Sprain |  |
| 4. Muscle Strain |  |
| 5. Neuropathy |  |
| 6. Vertebral disc involvement |  |
| 7. Other: Please specify: |  |

1. **During your current clinical rotation, how long do you spend doing the following activities per day? Please mark all that apply**

| ACTIVITIES | HOURS |
| --- | --- |
| 1.Lifting |  |
| 2.Transfers |  |
| 3.Bending |  |
| 4.Sitting |  |
| 5.Standing |  |

1. **Which ward activity were you doing at the first onset of your LBP?**  Please tick all that apply.

| 1.Giving Medication |  |
| --- | --- |
| 2. Bending or Twisting |  |
| 3. Instructing a patient |  |
| 4. Lifting |  |
| 5. Maintaining a position for a prolonged period. Please specify the posture. E.g. standing, sitting, kneeling, or bent over |  |
| 6. Performing repetitive tasks |  |
| 7. Responding to an unanticipated or sudden movement by a patient |  |
| 8. Transferring a patient |  |
| 9. Working in an awkward or cramped position |  |
| 10. Working when physically fatigued |  |
| 11. Pushing or pulling |  |
| 12. Other. Please specify: |  |

1. In the below table are 16 circumstances that may apply in your typical workday. On a scale of 0-5; (0=no problem, 1=minimal problem, 2=minor problem, 3=moderate problem, 4=severe problem, 5=very severe problem); please indicate to what extent each factor may have contributed to the development of your current low back pain.

| **RISK FACTOR** | **0** | **1** | **2** | **3** | **4** | **5** |
| --- | --- | --- | --- | --- | --- | --- |
| 1. Performing the same task over and over |  |  |  |  |  |  |
| 2. Working in the same position for long periods of time |  |  |  |  |  |  |
| 3. Working without shifts |  |  |  |  |  |  |
| 4. Bending or twisting your back in an awkward way |  |  |  |  |  |  |
| 5. Lifting or transferring dependent patients |  |  |  |  |  |  |
| 6. Continuing to work when injured or hurt |  |  |  |  |  |  |
| 7. Reaching or working away from your body |  |  |  |  |  |  |
| 8. Working in awkward or cramped positions |  |  |  |  |  |  |
| 9. Working near to or to your physical limits. |  |  |  |  |  |  |
| 10. Not enough rest breaks during the day |  |  |  |  |  |  |
| 11. Unanticipated sudden movement or fall by a patient |  |  |  |  |  |  |
| 12. Assisting patient during gait activities |  |  |  |  |  |  |
| 14. Working with confused or agitated patients |  |  |  |  |  |  |
| 15. Work schedule (e.g. overtime, on-call) |  |  |  |  |  |  |
| 16. Inadequate training in injury prevention |  |  |  |  |  |  |
| 17. Other (specify)……… |  |  |  |  |  |  |

1. Has your own experience with low back pain influenced your intent to specialize in a particular medical discipline? No Yes
2. If yes, which discipline(s) would you not consider specializing in based on your low back pain experience during clinical rotations? (name all that apply) . ……………………………………………………………….
